# An ethnobotanical survey of indigenous medicinal plants in Hafizabad district, Punjab-Pakistan

**DOI:** 10.1371/journal.pone.0177912

**Published:** 2017-06-02

**Authors:** Muhammad Umair, Muhammad Altaf, Arshad Mehmood Abbasi

**Affiliations:** 1 School of Agriculture and Biology and Research Center for Low-Carbon Agriculture Shanghai Jiao Tong University, Shanghai, China; 2 Department of Zoology, Women University of Azad Jammu and Kahmir, Bagh, Pakistan; 3 Department of Environment Sciences, COMSATS Institute of Information Technology, Abbottabad, Pakistan; Missouri Botanical Garden, UNITED STATES

## Abstract

Present paper offers considerable information on traditional uses of medicinal plants by the inhabitants of Hafizabad district, Punjab-Pakistan. This is the first quantitative ethnobotanical study from the area comprising popularity level of medicinal plant species intendedby using relative popularity level (RPL) and rank order priority (ROP) indices.Ethnobotanical data were collected by interviewing 166 local informants and 35 traditional health practioners (THPs) from different localities of Hafizabad district. Demographic features of informants; life form, part used, methods of preparation, modes of application and ethnomedicinal uses were documented. Ethnobotanical data were analyzed using quantitative tools, i.e. Relative frequency citation (RFC), use value (UV), informant consensus factor (ICF) fidelity level (FL), RPL and ROP indices. A total of 85 species belonging to 71 genera and 34 families were documented along with ethnomedicinal uses. *Solanum surattense*, *Withania somnifera*, *Cyperus rotundus*, *Solanum nigrum* and *Melia azedarach* were the most utilized medicinal plant species with highest used value. The reported ailments were classified into 11 disease categories based on ICF values and highest number of plant species was reported to treat dermatological and gastrointestinal disorders. *Withania somnifera* and *Ranunculus sceleratus* with maximum FL (100%), were used against gastrointestinal and urinary disorders, respectively. The RPL and ROP values were calculated to recognize the folk medicinal plant wealth; six out of 32 plant species (19%) were found popular, based on citation by more than half of the maximum number of informant viz. 26. Consequently, the ROP value for these species was more than 75. The comparative assessment with reported literature revealed 15% resemblance and 6% variation to previous data;however79% uses of the reported species were recorded for the first time. The diversity of medicinal plant species and associated traditional knowledge is significant in primary health care system. Medicinal plant species with high RPL values should be screened for comprehensive phytochemical and pharmacological studies. This could be useful in novel drug discovery and to validate the ethomendicinal knowledge.

## Introduction

Ethnomedicinal studies are of significant value to discover contemporary drugs from indigenous medicinal plant resources. There are appropriate sources of information about useful medicinal plant species, which can be targeted for management and domestication [1,2]. The documentation of traditional knowledge of native plant species has contributed a number of vital drugs [3,4]. Currently, 25% of herbal drugs in modern pharmacopeia are plant based and several synthetic drugs are manufactured by using chemical substances isolated from plants [5]. The fundamental role of natural products in the development of new drugs has been reported [6–8]. In recent era, the role of medicinal plant species in traditional health practice has diverted the attention of researchers towards ethnomedicines.

The use of plant species as traditional medicines provides a real substitute in healthcare services for rural communities of the developing nations [9]. It has been estimated that around 80% of the population in developing countries depends on traditional medicines for primary health care system. These traditional medicines are cost-effective, safe and affordable [5]. Globally, approximately 85% of the traditional medicines used in primary healthcare are derived from plantspecies [10]. Therefore, medicinal plants are the indigenous heritage of global importance [11]. Around, 50,000 flowering plants are used as medicinal [12], out 422,000 reported species of flowering plants [13]. At present, research on traditional uses of plant species has attained notableattention in the scientific community. Various workers have reported indigenous knowledge of medicinal plants from different parts of Pakistan [2,4,14–20], and few reports have been published in recent years [21–26].

The documentation of information on traditional herbal remedies is an important aspect of conservation approach. The present study, therefore, documents the traditional knowledge of local communities of Hafizabad district on medicinal uses of surrounding plant diversity. This work, being the first collation and listing of all available data on medicinal plants in this area, provides first ethnomedicinal and cultural assessment of these species. Specifically, present study was aimed: (i) to document the medicinal flora and traditional knowledge of local communities on indigenous plants used for medicinal purposes, (ii) to compile data on traditional treatments against various ailments, including method of preparation, plant part(s) utilization and application; (iii) to evaluate the ethnomedicinal data using RPL and ROP indices in order to explore most popular species, which could be useful for in depth pharmacological screening.

## Materials and methods

### Description of the study area

**Fig 1:**
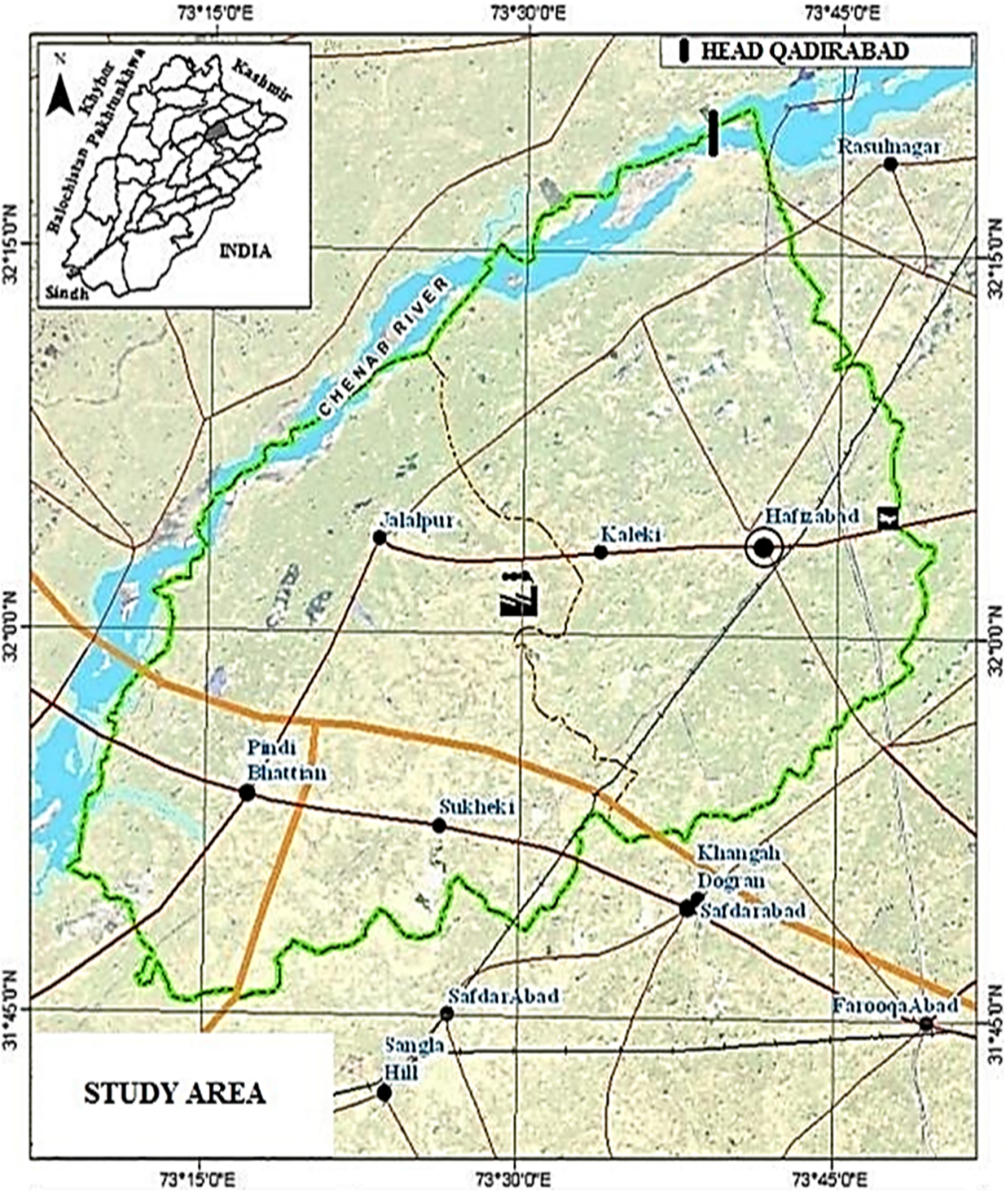
Map of district Hafizabad-Pakistan.

The present study was conducted to document the traditional knowledge of local communities on medicinal plant species from six localities i.e. Kaleke Mandi, Head Qadirabad, Hafizabad city, Sukhakee, Pindi Bhattian and Jalalpur of district Hafizabad, Punjab province-Pakistan (Fig 1). The study area lies between 32° 20′N and 73° 46′E, surrounded by Sheikhupura on south, Sargodha on west, Gujranwala on east and by Mandi Bahuadd in on north-west. The survey site spreads an area of 2,367 km^2^ along with altitudinal variation of 207m above sea level. The forest cover is approximately 550.4ha (0.23%) of the total of the district. The Chenab River spreads from north to north west of the district. Climate of the study area falls in semi-arid type with temperature ranged from 48^°^C (in summer) to 1^°^C (in winter). The study area receives highest rainfall during monsoon (July to September). The annual rainfall and humidity varies from 50–70mm and 25–85%, respectively [27,28]. According to Punjab development statistics 2011, total human population of the study area was 1,038,000, which includes: 52% males and 48% females. Around 27.26% of the population lives in urban areas, while remaining 72.74% comprises rural communities. The major ethnic group speaks Punjabi (98.7%), while other languages spoken are Urdu (0.8%), Pushto (0.4%) and Siraiki (0.1%).

### Data collection and identification of plant species

Semi structured interviews and group conversation with local inhabitants were conducted to gather information on medicinal uses of plant species from 201 informants including male, female respondents and traditional healers. Data on age, gender, educational status and linguistics of respondents were also gathered. Information regarding the local plant names, part(s) used, methods of preparation and application were documented. The medicinal plant species used by the local communities of the study area were authenticated using the international plant name index (http://www.ipni.org), the plant list (www.theplantlist.org) and GRIN taxonomy site (http://www.ars-grin.gov/cgi-bin/npgs/html/queries.pl), while that of families follow A.P.G. system [29]. The species entries were complemented along with data on taxonomic position (family), vernacular name, common name, flowering period, life form and folk medicinal uses. The life form was categorized into herbs, shrubs, grasses and trees (annual, biennial or perennial), according to the system proposed by Raunkiær [30,31] and modified by Brown [32]. Collected plant species were identified by Prof. Dr. Rizwana Aleem Qureshi (Plant Taxonomist, Quaid-i-Azam University, Islamabad and by using Flora of West Pakistan [33] and Flora of Punjab [34]. The voucher specimens were submitted in Herbarium of Pakistan (ISL) Quaid-i-Azam University Islamabad.

### Quantitative analysis

The ethnobotanical data was analyzed using different quantitative indices including Informant Consensus Factor (ICF), Use value (UV), Relative frequency citation (RFC), Fidelity level (FL), Relative popularity level (RPL), Rank order priority (ROP). Data were reported in proportions and percentages.

#### Informant consensus factor (ICF)

ICF value describes informants’ consensus on the medicinal plant consumption species, and evaluates variability in mode of utilization against reported diseases. Before calculating ICF value, ailments are broadly categorized into different categories [35]. The maximum ICF value i.e. close to 1 indicates that well known species are used by a large proportion of local communities due to their authenticity regarding diseases. However, low ICF index close to 0 specifies that the informants use this species randomly to treat reported diseases[35].

The ICF value was calculate using the formula as described earlier [36,37]:

$$ICF=\frac{N_{ur}-N_{t}}{N_{ur}-1}$$

Where, “N_*ur*_^”^ is the total number of use reports for each disease category and “N_*t*_” indicates the number of species used in said category.

#### Use value (UV)

Use value (UV) determines the relative importance on uses of plant species. It is calculated using the following formula as explained before [37,38]:

$${UV}_{i}=\frac{\Sigma U_{i}}{N}$$

Where, “UV” indicates use value of individual species, “U” is the number of uses recoded for that species and “N” represents the number of informants who reported that species.

#### Relative frequency of citation (RFC)

Relative frequency of citation(RFC)signifies the local importance of each species in a study area [39,40]. This index is determined by dividing the number of informants citing a useful species (FC) by total number of informants in the survey (N). RFC is calculated by the formula as described earlier [41]:

$$RFC=\frac{FC}{N}\;(0<RFC<1)$$

#### Fidelity level (FL)

FL is the percentage of informants who mentioned the uses of certain plant species to treat a particular ailment in a study area. The FL index is calculated using formula as reported previously [42,43]:

$$FL\;(\%)=\frac{N_{P}}{N}\times 100$$

Where, ‘Np’ is the number of informants that claimed a use of certain plant species for a particular disease and ‘N’ is the total number of informants citing the species for any disease. The maximum FL indicates the frequency and high use of the plant species for treating a particular ailment by the informants of the study area.

#### Relative popularity level (RPL)

RPL is the ratio between number of ailments treated by a particular plant species and the total number of informants for any disease. However, plant species with comparable FL may vary in their healing potential. A correction scale is therefore introduced, in which all the encountered plant species are divided into popular and unpopular groups. The relative popularity level (RPL) assumes a value 0 and 1.0, with ‘1’ being complete popularity of a plant for major ailments and ‘0’ no ailments treated by a plant species. When all plant species are frequently used to treat some major ailments, popularity index would be maximum (1.0); then decrease towards zero as the relative popularity of the species diverge away from popular side. For popular plant species, the RPL value is rationally selected to equal unity (i.e. equal to 1), while RPL value is less than 1 for unpopular plant species. The relative popularity level (RPL) of the plant species is calculated and designated as popular or unpopular. The RPL value may be determined for each specific plant in accordance with its exact position on graph [43,44].

#### Rank order priority (ROP)

ROP is a correction factor, used for appropriate ranking of the plant species with different FL and RPL values. The ROP is derived from FL; by multiplying RPL and FL values as explained earlier [43,44].

ROP=FL×RPL

## Results and discussion

### Demographic features of the informants

**Fig 2:**
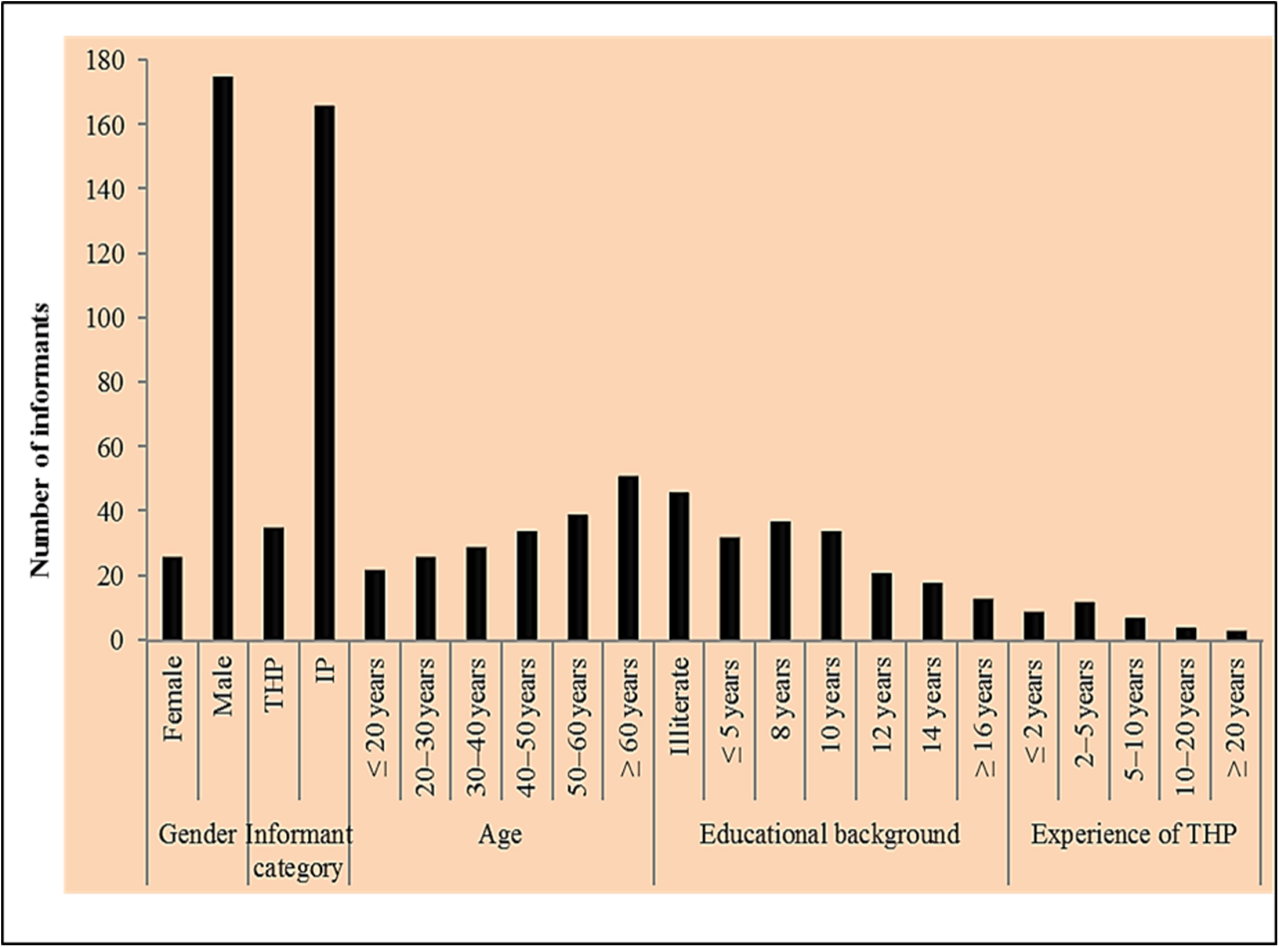
Demographic data of local informants (THPs. traditional health practioners, IP. indigenous peoples).

A total of 201 local informants including 175 males and 26 females were interviewed. Based on demography these informants were categorized into different classes as given in Fig 2. In the present survey male participants were higher than females. The prevalence of male informants is due to the fact that females of the study area were reluctant in conversation with male strangers (the interviewers). The local informants were farmers, foresters or herdsmen, craftsmen, shopkeepers, teachers and housewives. The traditional health practioners (THPs) hold significant information on the medicinal uses of local plant species to treat various ailments. THPs were classified into five groups on the basis of their experience such as THPs having less than 2 years’ experience (9), THPs with 2–5 years’ experience (12), THPs of 5–10 years’ experience (7), THPs of 10–20 years’ experience (4) and 3 THPs have more than 20 years’ experience. Among others, maximum informants having traditional knowledge regarding the use of medicinal plants were fall in secondary school education level or even below this and often spoke only Punjabi language. The maximum information was collected from the informants above 60 years age possess significant traditional knowledge whereas little information was shared by young respondents. Moreover, illiterate informants shared possess more information on the traditional use of medicinal plant species compared to educated respondents. This may be due to changing lifestyle, increase in the use of allopathic medicine and urbanization. Similar findings have also been reported from Bangladesh [45]and Turkey[9,46].

### Taxonomic classification

In total 85 medicinal plant species belonging to 71 genera and 34 families were documented(Table 1). All reported species were angiosperms, which include: monocotyledonous and dicotyledonous groups (10.6 and 89.4%, respectively). Asteraceae and Poaceae were the most dominant families (with 9 species each), followed by Fabaceae (8 species), Moraceae and Euphorbiaceae (6 species each), Chenopodiaceae (5 species), Malvaceae and Solanceae (4 species each), Amaranthaceae (3 species) and Meliaceae, Myrtaceae, Oleaceae, Polygonaceae and Rhamnaceae (2 species each), while other families were represented by one species only (S1 Fig) The utilization of medicinal plant species belonging to Asteraceae and Poaceae families was in agreement with ethnomedicinal flora reported from other parts of Pakistan and in other areas of the world [23,47–49]. This may be due to their wide distribution of plant species belonging to Asteraceae [50] and Poaceae [51] and their traditional uses known by the indigenous communities living in different parts of the world.

**Table 1:** Ethnomedicinal plant species of the study area.

S #.	Plant species with families and Accession number	Common Name	Local Name	Flowering Period	^A^ Life Habits/ Life forms			^B^ Part (s)/Mode of utilization	Application mode	Therapeutic uses	^C^RFC	^C^UV	^D^ Previously Used
1	*Achyranthes aspera* L. Amaranthaceae, HAF-01	Prickly-Chaff flower	Puthkanda	March-October	W	H	P	WP. decoction, extract; ST. powder; LE. paste, powder; RT. decoction; RT. juice	Oral, Topical and as Toothbrush	Pneumonia, kidney stone, ulcer, chest pain, external wounds, dysmenorrhea, toothache, asthma	0.11	0.43	26◆101∎21◆102●103∎104◆66●105∎80◆
2	*Amaranthus spinosus* L. Amaranthaceae, HAF-02	Spiny pigweed	Gnar	May-July	W	H	A	LE. cooked, juice, extract; RT. juice, decoction; SD. powder; BA. decoction	Oral, Gargle	Anthelminthic, stomachache, diuretic, cataract, toothache, constipation	0.09	0.32	26◆101∎21◆102●103∎104◆66●105∎80◆
3	*Amaranthus viridis* L. Amaranthaceae, HAF-03	Slender amaranth	Ganhar	March-October	W	H	A	LE. extract, cooked, juice. paste; SD. powder; RT. decoction	Oral, Topical	Cough and asthma, eye vision, constipation, file, painful urination, snakebite	0.08	0.35	26◆101∎21◆102●103∎104◆66●105∎80◆
4	*Mangifera indica* L. Anacardiaceae, HAF-04	Mango	Aam	March-April	C	T	P	LE and BA. latex; FR. juice; LE. decoction, paste. infusion; SD. extract	Topical, Oral	Heel cracks, diarrhea, fever, snake bite, diabetes, blood pressure	0.14	0.21	26◆101∎21◆102●103∎104◆66●105∎80◆
5	*Anethum graveolens* L.* Apiaceae, HAF-82	Dill	Sowa	May-July	W/C	H	A/P	LE. powder, infusion; SD. powder	Oral	Indigestion, flatulence, bronchitis	0.10	0.14	26◆101∎21◆102●103∎104◆66●105∎80◆◆
6	*Calotropis procera* Br. Asclepiadaceae, HAF-05	Milk weed	Akh	Throughout the year	W	S	P	LE. paste, extract, poultice. latex; ST. latex; ST and LE. decoction	Oral, Topical and as Inhale	Wound healing, asthma, toothache, T.B., hepatitis, burns, malarial fever, lice killer	0.13	0.31	26◆101∎21◆102●103∎104◆66●105∎80◆
7	*Ageratum conyzoides* L. Asteraceae, HAF-06	Goat weed	Knaar	July-September	W	H	A	LE. paste, juice, extract; FL. decoction; ST. powder; WP. juice; RT. juice	Oral, Topical and as Eye drop	Cut and wounds, fever, flu and cough, infertility, jaundice, hair tonic, conjunctivitis, stomachache	0.12	0.36	26◆101∎21◆102●103∎104◆66●105∎80◆
8	*Cirsium arvense* (L.) Scop* Asteraceae, HAF-07	Creeping thistle	Kandaal/Leh	May-August	W	H	P	LE. Juice; ST.FL.RT. decoction	Topical, Oral	Wounds, peptic ulcer, tonic, cough and bronchitis	0.06	0.33	26◆101∎21◆102●103∎104◆66●105∎80◆
9	*Conyza ambigua* DC.Asteraceae, HAF-08	Hairy fleabane	Gider buti	August-October	W	H	A/P	WP. Extract; RT. Decoction; LE. Infusion, juice	Oral	Diarrhea and dysentery, painful menstruation, bleeding piles, diabetes, hypertension	0.08	0.47	26◆101∎21◆102●103∎104◆66●105∎80◆
10	*Coronopus didymus* (L.) Sm. Asteraceae, HAF-09	Swine-cress	Jangli haloon	May-August	W	H	A/B	ST. powder; LE. infusion; WP. Juice; SH. Extract; FL. decoction	Oral, Topical	Bone fracture, tumors, rheumatism, blood purifier, nerve tonic, cold, fever and flu	0.13	0.33	26◆101∎21◆102●103∎104◆66●105∎80◆
11	*Eclipta alba* (L.) Hassk Asteraceae, HAF-10	Trailing ecliptic Plant	Sofed banghara	July-October	W	H	P	WP. Powder, poultice, decoction; LE. powder, juice/tea; RT. decoction	Oral, Topical	Malarial fever, burns, blood purifier, burning urination, liver cancer, hair tonic	0.15	0.37	26◆101∎21◆102●103∎104◆66●105∎80◆
12	*Launaea procumbens* Roxb. Asteraceae, HAF-12	Creeping-launaea	Pili dodhak	March-August	W	H	P	LE. extract, powder, paste; WP. infusion	Oral, Topical	Dysuria, ringworm, fever, galactagogue	0.10	0.20	26◆101∎21◆102●103∎104◆66●105∎80◆
13	*Parthenium hysterophorus* L* Asteraceae, HAF-14	Feverfew	Gandi boti	April-August	W	H	A/P	LE. juice, extract; WP. juice, decoction; FL. Powder; RT. Juice	Oral	Constipation, toothache, vermifuge, diabetes, emmenagogue, tonic	0.05	0.60	26◆101∎21◆102●103∎104◆66●105∎80◆
14	*Sonchus asper* Hill. Asteraceae, HAF-11	Spiny leaved so whistle	Asgandh	May-July	W	H	A	LE.RT. SH. decoction; LE. paste; WP. powder	Oral, Topical	Fever, cough, asthma, constipation, wounds, dyspepsia	0.11	0.27	26◆101∎21◆102●103∎104◆66●105∎80◆
15	*Xanthium strumarium* L. Asteraceae, HAF-13	Cocklebur	Chhota dhatura	May-July	W	H	A	LE. decoction, powder; FR. decoction; RT. Powder	Oral, Topical and as Toothbrush	Malarial fever, skin burn, paralysis, stomachache, small pox, scrofulous tumors, dental soreness	0.07	0.47	26◆101∎21◆102●103∎104◆66●105∎80◆
16	*Brassica rapa* L. Brassicaceae, HAF-16	Field mustard	Sarsoon	January-March	C	H	B	LE. decoction; WP. cocked; SD. powder	Oral, Topical	Skin edema, blood purifier, tonic	0.09	0.16	26◆101∎21◆102●103∎104◆66●105∎80◆
17	*Sisymbrium irio* L. Brassicaceae, HAF-15	London rocket	Khoob kalan	March-May	W	H	A	WP. juice; FR. infusion; decoction, powder; SD. poultice	Oral, Topical	Ophthalmia, mumps and measles, dyspepsia, face pimples, cuts and wounds	0.11	0.22	26◆101∎21◆102●103∎104◆66●105∎80◆
18	*Cassia fistula* L. Caesalpiniaceae, HAF-17	Golden shower	Amaltas	April-June	W	T	P	FR. BA. Powder; FL. ash; LE. decoction, paste; RT. powder	Oral, Topical	Constipation, gastric, jaundice, cough, urinary tract infection, eczema, wounds, rheumatism	0.10	0.38	26◆101∎21◆102●103∎104◆66●105∎80◆
19	*Capparis deciduas* (Forssk.) Edgew Capparidaceae, HAF-18	Caper plant	kerda, kair	April-July	W	T	P	FL.ST.RT. powder; FR. BA. powder; SH. decoction; LE. paste; FL.SD. decoction	Oral, Topical	Male sexual dysfunction, hemolytic anemia, vermifuge, stomachache, liver tonic, boils and swellings, sciatic	0.05	0.64	26◆101∎21◆102●103∎104◆66●105∎80◆
20	*Stellaria media* L. Caryophyllaceae, HAF-19	Chickweed	Gandhar	April-August	W	H	A	WP. decoction; SD. LE. paste, extract, poultice	Oral, Topical	Regular bowl, itchy skin, wounds, swelling joints, bone fracture	0.11	0.23	26◆101∎21◆102●103∎104◆66●105∎80◆
21	*Chenopodium album* L. Chenopodicaeae, HAF-20	Lamb’s quarter	Bathu	January-September	W/C	H	A	LE. juice, infusion; WP. cooked; RT. decoction; SH.FL. juice	Oral	Laxative, gastritis, hepatic and urinary disorder, rheumatism, constipation, vermifuge	0.11	0.74	26◆101∎21◆102●103∎104◆66●105∎80◆
22	*Chenopodium ambrosioides* L. Chenopodicaeae, HAF-21	Sweet pigweed	Chandanbathwa	April-February	W	H	A/P	FL. juice; LE. infusion, powder, decoction; WP. juice; SH.FL. juice	Oral, Topical	Hypertension, menstrual disorders, hemorrhoids, toothache, constipation, stomachache	0.10	0.35	26◆101∎21◆102●103∎104◆66●105∎80◆
23	*Chenopodium murale* L. Chenopodicaeae, HAF-22	Australian-spinach	Karund	January-July	W	H	A	WP. decoction; LE.ST. paste; LE. powder, decoction; SD. powder +	Oral, Topical and as Snuff	Stomachache, lameness, cough and cold, infertility, vermifuge	0.08	0.29	26◆101∎21◆102●103∎104◆66●105∎80◆
24	*Kochia indica* Wight* Chenopodicaeae, HAF-24	Indian bassia	Boi	July-October	W	H	A/B	FR; LE. decoction, oil	Oral, Gargle	Heart tonic, diuretic, toothache,	0.04	0.38	26◆101∎21◆102●103∎104◆66●105∎80◆
25	*Suaeda fruticosa* Forsk. Chenopodicaeae, HAF-23	Seep weed	Khaari	April-September	W	S	P	LE. decoction, juice; ST. decoction, ash; WP. decoction	Oral, Topical	Diuretic, blood purifier, liver cancer, snake bites, kidney stone, hair tonic	0.08	0.41	26◆101∎21◆102●103∎104◆66●105∎80◆
26	*Convolvulus arvensis* L. Convolvulaceae, HAF-25	Deer’s foot.	Lehli/Vahri	Throughout the year	W	H	A/P	RT.WP. cooked, extract; LE. juice, paste	Oral, Topical	Constipation, blood purification, rheumatic pain, hair tonic, skin ulcer	0.08	0.38	26◆101∎21◆102●103∎104◆66●105∎80◆
27	*Cyperus rotundus* L. Cyperaceae, HAF-26	Nut grass	Daila	April-July	W/C	H	P	RT. infusion; LE. decoction, paste; RH. powder, paste, decoction	Oral, Topical	Diuretic, vermifuge, dermatitis, stomachache, galactagogue, hypersplenism	0.12	0.84	26◆101∎21◆102●103∎104◆66●105∎80◆
28	*Chrozophora tinctoria* (L.) A.Juss* Euphorbiaceae, HAF-27	Giradol	Neeli Booti	January-September	W	H	A	LE. juice, decoction, extract; ST. juice	Oral, Eye drop	Stomachache, sore throat, emetic, cataract	0.07	0.20	26◆101∎21◆102●103∎104◆66●105∎80◆
29	*Croton sparsiflorus* Morong Euphorbiaceae, HAF-32	Herb pimento	Ban tulsi	April-July	W	H	P	LE. poultice, decoction, juice; RT. powder; ST. juice; WP. juice, decoction	Oral, Topical	Bone fracture, gastric ulcer, hemorrhage, hair tonic, dermatitis, dengue fever, cardiac tonic	0.05	0.64	26◆101∎21◆102●103∎104◆66●105∎80◆
30	*Euphorbia dracunculoides* Lam. Euphorbiaceae, HAF-31	Dragon spurge	Bamburi	November-April	W	H	A/P	LE. juice, paste, powder; FR. juice	Oral, Topical	Lice killer, headache, skin parasites, snake bite, epilepsy	0.09	0.28	26◆101∎21◆102●103∎104◆66●105∎80◆
31	*Euphorbia helioscopia* L. Euphorbiaceae, HAF-28	Sun euphorbia	Chhatri dodak	January-July	W	H	A	RT.WP. juice, latex, powder; SH; SD	Oral, Topical, Eye drop	Anthelminthic, athlete’s foot, eye sores, asthma, constipation, cholera	0.11	0.39	26◆101∎21◆102●103∎104◆66●105∎80◆
32	*Euphorbia pilulifera* L. Euphorbiaceae, HAF-29	Asthma weed	Doddak	July-December	W	H	A	LE. juice; FL and SD. powder; WP. decoction, latex, juice	Oral, Topical and as Eye drop	Asthma, cough, stomachache, diarrhea, eye redness, burns, cut and warts	0.09	0.39	26◆101∎21◆102●103∎104◆66●105∎80◆
33	*Euphorbia prostrate* L* Euphorbiaceae, HAF-30	Creeping spurge	Doodi buti	Throughout the year	W	H	P	WP. extract, latex, decoction; LE. infusion	Oral, Topical	Diarrhea and dysentery, liver tonic, ringworm, blood purifier, diabetes, kidney stone	0.12	0.29	26◆101∎21◆102●103∎104◆66●105∎80◆
34	*Acacia modesta* Wall. Fabaceae, HAF-42	Amritsar gum	Phulai	March-May	W	T	P	BA. powder; ST. gum, extract; LE. extract; LE. ST. latex; BA. ass	Oral, Topical, Toothbrush	Toothache, carminative, tonic, cooling agent, back pain, bronchitis, asthma	0.06	0.54	26◆101∎21◆102●103∎104◆66●105∎80◆
35	*Acacia nilotica* L. Fabaceae, HAF-41	Babul acacia	Kikar	March-August	W	T	P	BA. powder, decoction; LE. decoction, paste; ST. gum; BA. ash; LE. paste; FL. powder	Oral, Anal and as Toothbrush	Hyperglycemia, stomach-ache, dysentery, backbone and joints pain, toothache, piles, jaundice	0.10	0.38	26◆101∎21◆102●103∎104◆66●105∎80◆
36	*Alhagi maurorum* Medik. Fabaceae, HAF-58	Camel thorn	Jawansa	April-September	W	S	P	RT. decoction; FR. powder; LE. decoction; RT. infusion; WP. decoction; SD. powder	Oral, Topical	Kidney stone, bleeding piles, constipation, cough, asthma, blood purifier, rheumatic pain, burn	0.07	0.53	26◆101∎21◆102●103∎104◆66●105∎80◆
37	*Dalbergia sissoo* Roxb. Fabaceae, HAF-57	Indian rose wood	Tali	March-May	W	T	P	LE. decoction, paste, infusion; BA. decoction; BA. powder;	Oral, Topical	Emetic, diarrhea and vermifuge, cut and wounds, fever, nosebleed, constipation, hair tonic	0.08	0.44	26◆101∎21◆102●103∎104◆66●105∎80◆
38	*Pongamia pinnata* (L.) Pierre Fabaceae, HAF-56	Pongam oil tree	Sukh chain	April-May.	C	T	P	ST; LE. powder; SD. oil; BA. decoction; FL. powder; RT. juice	Oral, Topical	Toothache, carminative, rheumatism, vermifuge, diabetes, wounds and ulcer	0.08	0.35	26◆101∎21◆102●103∎104◆66●105∎80◆
39	*Prosopis cineraria* L. Fabaceae, HAF-43	*Prosopis*	Jhand	December-March	W/C	S	P	LE. juice, paste; BA. powder; ST. decoction; FL. powder; FR. paste, powder	Oral, Topical, Eye drop	Liver tonic, boils and blisters, scorpion bite, pancreatic stone, leucorrhoea, chronic dysentery, cataract	0.09	0.42	26◆101∎21◆102●103∎104◆66●105∎80◆
40	*Prosopis juliflora* Swartz* Fabaceae, HAF-40	Honey mesquite	Mosquit pod	March-June	W	T	P	WP. decoction; FL. infusion; ST.LE. juice, poultice; BA. powder	Oral, Topical and as Toothbrush	Galactagogue, kidney stones, toothache, breast cancer, asthma, boils	0.07	0.40	26◆101∎21◆102●103∎104◆66●105∎80◆
41	*Trifolium resupinatum* L* Fabaceae, HAF-55	Reversed clover	Loosin	April-August	W	H	A	WP. decoction, infusion; FL. powder	Oral, Gargle	Sore throat, cough, skin sores, ulcer, sedative, liver tonic, digestion	0.09	0.39	26◆101∎21◆102●103∎104◆66●105∎80◆
42	*Oxalis corniculata* L. Geraniaceae, HAF-33	Clover sorrel	Khatti buti	March-December	W	H	P	LE. cocked, paste, cooked; WP. decoction, powder; RT. decoction	Oral, Topical and as Eye drop	Diarrhea and dysentery, wounds, hepatitis and jaundice, eye ache, anthelminthic, premature ejaculation	0.09	0.37	26◆101∎21◆102●103∎104◆66●105∎80◆
43	*Hibiscus rosa-sinensis* L. Malvaceae, HAF-37	Rose mallow	Gurhal	Throughout the year	C	S	P	FL. powder, juice; LE. paste, juice, tea; RT. powder	Oral, Topical	Sexual dysfunction, eczema, heart burn, loss of appetite, asthma, regular bowl, leucorrhoea	0.09	0.74	26◆101∎21◆102●103∎104◆66●105∎80◆
44	*Malva parviflora* L. Malvaceae, HAF-34	Cheese-weed	Sonchal	May-September	W	H	A	SD. decoction; SH.SD. decoction; SH. LE. decoction, extract, poultice	Oral, Topical	Cough and bronchitis, abortifacient, throat-ache, cough, fever, constipation, scorpion sting	0.11	0.32	26◆101∎21◆102●103∎104◆66●105∎80◆
45	*Malvastrum tricuspidatum* A. Gray * Malvaceae, HAF-35	False mallow	DhamniButi	December-August	W	H	A	LE. poultice, paste, decoction; WP. powder	Oral, Topical	Sores and wounds, eczema, asthma, diarrhea	0.04	0.44	26◆101∎21◆102●103∎104◆66●105∎80◆
46	*Malvaviscus arboreus* (Torr. & Gray) Schery Malvaceae, HAF-36	Sleeping hibiscus	Max mallow	Throughout the year	C	S	P	FL. decoction, infusion; LE. decoction, juice	Oral, Topical	Sore throat, diarrhea, fever, lice killer	0.03	0.67	26◆101∎21◆102●103∎104◆66●105∎80◆
47	*Azadirachta indica* A. Juss. Meliaceae, HAF-39	Neem	Neem	April-May	W/C	T	P	LE. decoction, infusion, paste; SD. oil; ST.BA. decoction; LE. paste	Oral, Topical, Toothbrush	Blood purifier, diabetes, malaria, intestinal worm, headache, toothache, liver tonic, rheumatism, small pox	0.13	0.33	26◆101∎21◆102●103∎104◆66●105∎80◆
48	*Melia azedarach* L. Meliaceae, HAF-38	Chinaberry	Dhraikh	May-July	W/C	T	P	LE. decoction, juice, paste, extract, infusion; BA. powder; ST. decoction	Oral, Topical, Bath	Malarial fever, allergy, wound healing, blood purifier, urinary stones, high blood pressure, diabetes	0.07	0.79	26◆101∎21◆102●103∎104◆66●105∎80◆
49	*Ficus benjamina* L* Moraceae, HAF-44	Weeping fig	Kabar	October-January	W	T	P	ST. decoction; WP. powder; LE. decoction; FR.BA and LE. cocked	Oral, Topical	Stomachache, blood purification, ulcers, carminative, joint and back pain	0.06	0.42	26◆101∎21◆102●103∎104◆66●105∎80◆
50	*Ficus racemosa* L. Moraceae, HAF-45	Cluster tree	Gular	March-May	W/C	T	P	ST. latex; LE. juice; BA. FR. powder, decoction	Oral, Topical, Anal	Piles, diarrhea, obesity, carminative, boils and ulcer	0.09	0.37	26◆101∎21◆102●103∎104◆66●105∎80◆
51	*Ficus religiosa* L. Moraceae, HAF-46	Sacred fig	Pipal	March-October	W	T	P	LE. decoction, paste, infusion; FR. powder; ST. powder; RT. extract	Oral, Topical	Body pain, asthma, gonorrhea, skin ulcer, heart blockage, diabetes	0.11	0.27	26◆101∎21◆102●103∎104◆66●105∎80◆
52	*Ficus virens* L. Moraceae, HAF-47	White fig	Palakh	October-March	W	T	P	FR. powder; BA. infusion; ST. latex	Oral	Diabetes, ulcer, breast cancer	0.06	0.25	26◆101∎21◆102●103∎104◆66●105∎80◆
53	*Morus alba* L. Moraceae, HAF-48	White mulberry	Shahtoot	April-September	C	T	P	FR. decoction, juice; ST. latex; WP. decoction; LE. juice; BA and LE. decoction	Oral, Topical	Constipation, cough, liver tonic, tonsils, snakebite, diabetes	0.11	0.74	26◆101∎21◆102●103∎104◆66●105∎80◆
54	*Morus nigra* L* Moraceae, HAF-49	Black mulberry	Kala toot	March-July	C	T	P	WP. decoction; FR. juice, decoction; LE. decoction, infusion; RT. powder	Oral, Gargle	Asthma, throat ache, cough, flu, toothache, constipation, carminative, intestinal worms, diabetes	0.13	0.74	26◆101∎21◆102●103∎104◆66●105∎80◆
55	*Eucalyptus camaldulensis* (A.) Cunn. Myrtaceae, HAF-51	River red-gum	Safaida	April-October	W	T	P	LE. decoction; juice, extract	Oral, Gargle	Common cold, sinusitis, sore throat, cough, flu, fever	0.10	0.29	26◆101∎21◆102●103∎104◆66●105∎80◆
56	*Psidium guajava* L. Myrtaceae, HAF-50	Guava	Amrud	March-April	C	S	P	FR.LE. infusion, decoction, extract; FL. decoction	Oral, Gargle	Diarrhea, diabetes, diuretic, carminative and vermifuge, toothache, fever, flu, cough	0.11	0.78	26◆101∎21◆102●103∎104◆66●105∎80◆
57	*Boerhavia diffusa* L. Nyctaginaceae, HAF-52	Horse-purslane	Itsit	September-August	W	H	A/P	WP. infusion; LE. paste; RT. decoction, powder	Oral, Topical	Dysmenorrheal, snakebite, kidney failure, cough, flu and asthma	0.10	0.29	26◆101∎21◆102●103∎104◆66●105∎80◆
58	*Jasminum officinale* L* Oleaceae, HAF-53	Poet's jasmine	Malti	May July	C	S	P	ST. juice, extract; FL. decoction; LE. sap, extract; WP. extract	Oral, Topical	High fever, intestinal worms, conjunctivitis, diarrhea, scabies, heart burn and cough	0.07	0.73	26◆101∎21◆102●103∎104◆66●105∎80◆
59	*Jasminum sambac* (L.)Ait. Oleaceae, HAF-54	Arabian jasmine	Motia	April-June	C	S	P	FL. juice; LE. paste, decoction, juice, extract; RT. decoction	Oral, Topical	Conjunctivitis, wound and acne, insomnia, emmenagogue, ulcer, breast tumors, high fever	0.05	0.64	26◆101∎21◆102●103∎104◆66●105∎80◆
60	*Cenchrus pennisetiformis* Hoschst. & Steud. Poaceae, HAF-60	White buffalo grass	Cheetah gha	February-April	W	G	A/P	LE. extract, juice, infusion; FR. decoction; ST. juice	Oral, Topical	Asthma, T.B., Cough, bleeding piles, epilepsy, skin irritation, eczema	0.07	0.50	26◆101∎21◆102●103∎104◆66●105∎80◆
61	*Cynodon dactylon* Pers. Poaceae, HAF-61	Bermuda grass	Khanbalgha	May-September	W	G	P	RH. oil, decoction WP. decoction, paste, juice; RT. infusion	Oral, Topical and as Eardrop	Hypertension, kidney stones, itching, eye pain, earache, indigestion	0.07	0.73	26◆101∎21◆102●103∎104◆66●105∎80◆
62	*Dactyloctenium aegyptium* Beauv. Poaceae, HAF-62	Crow’s foot grass	Madhanagha	July-October.	W	G	A	SD; RT; WP. paste	Oral, Topical	Kidney stones, uterus problem, stomachache, wounds and ulcer	0.04	0.67	26◆101∎21◆102●103∎104◆66●105∎80◆
63	*Dicanthium annulatum* Stapf. Poaceae, HAF-63	Ringed dichanthium	Murgha gha	March-November	W	G	P	LE. ST. decoction; LE. juice, infusion, paste; ST. powder	Oral, Topical	Abortifacient, diarrhea, indigestion, piles, antispasmodic, scabies	0.06	0.50	26◆101∎21◆102●103∎104◆66●105∎80◆
64	*Eleusine indica* (L.) Gaertn. Poaceae, HAF-64	Goose grass	Madhani	June-August	W	G	A	RH. Extract; RT. powder; LE. juice; WP. infusion, tea, decoction	Oral, Topical	Fever, dysentery, prolapse of uterus, diabetes, food poisoning, hair loss	0.04	0.75	26◆101∎21◆102●103∎104◆66●105∎80◆
65	*Imperata cylindrical* L. Poaceae, HAF-65	Cogon grass	Dabh gha	March-November	W	G	P	LE. paste; RH. decoction; LE.SH. paste; RT. decoction	Oral, Topical	Tonic, cut and wounds, urodynia, hypertension, febrifuge	0.04	0.56	26◆101∎21◆102●103∎104◆66●105∎80◆
66	*Setaria glauca* Beauv* Poaceae, HAF-66	Yellow foxtail	Bajra	May-October	W	G	A/P	ST. decoction; LE.ST.SD juice, infusion	Topical	Wound healing, dermatitis, ring worm, tonic, hair tonic	0.03	0.71	26◆101∎21◆102●103∎104◆66●105∎80◆
67	*Sorghum halepense* Pers* Poaceae, HAF-67	Johnson grass	Baru/Lamjack	May-October	W	G	P	RT. decoction; SD. powder; ST. juice	Oral, Topical	Indigestion, cough, boils, demulcent	0.04	0.50	26◆101∎21◆102●103∎104◆66●105∎80◆
68	*Triticum aestivum* L. Poaceae, HAF-59	Wheat	Kanak	April-May	C	G	A	SH. decoction; SD. decoction, paste, powder; RT. decoction	Oral, Topical	Colon cancer, anemia, wound healing, late puberty, asthma, diabetes	0.15	0.77	26◆101∎21◆102●103∎104◆66●105∎80◆
69	*Polygonum plebejum* R. Br. Polygonaceae, HAF-68	Small knotweed	Hind rani	March-June	W	H	A	WP. paste, powder, RT. decoction, SH. decoction, LE. extract	Oral, Topical	Eczema, galactagogue, pneumonia, liver-tonic, heartburn, regular bowl	0.10	0.35	26◆101∎21◆102●103∎104◆66●105∎80◆
70	*Rumex dentatus* L. Polygonaceae, HAF-69	Toothed dock	Jangli palak	May-June	W	H	A	RT. powder, decoction; RH and LE. poultice; WP. decoction	Oral, Topical	Constipation, cuts and wounds, eczema, cooling agent	0.05	0.64	26◆101∎21◆102●103∎104◆66●105∎80◆
71	*Anagallis arvensis* L. Primulaceae, HAF-70	Scarlet pimpernel	Bili booti	March-April	W	H	A	WP. juice, pate; FL.LE. decoction; ST. powder	Oral, Topical	Leprosy, skin ulcer, epilepsy, hepatitis	0.09	0.22	26◆101∎21◆102●103∎104◆66●105∎80◆
72	*Ranunculus sceleratus* L* Ranunculaceae, HAF-71	Blister buttercup	Gul-e-ashrafi	March-April	W	H	A/B	WP. decoction, juice, infusion; SD; RT. paste	Oral, Topical	Asthma and fever, tonic, muscle hamstring, vermifuge, urinary incontinence	0.14	0.25	26◆101∎21◆102●103∎104◆66●105∎80◆
73	*Ziziphus nummularia* (Burm. f.) Wight &Arn. Rhamnaceae, HAF-73	Jujube	Bair	March-June	W	S	P	LE. decoction, paste; FR. powder; BA. decoction	Oral, Topical	Tonic, diabetes, constipation, cold and sore throat, scabies	0.09	0.26	26◆101∎21◆102●103∎104◆66●105∎80◆
74	*Zizyphus mauritiana* Lam. Rhamnaceae, HAF-72	Chinese apple	Bairi	July-September	W	T	P	LE.BA. decoction; LE. extract, decoction, juice; BA. powder; RT. decoction;	Oral, Topical, Bath, Gargle	Chicken pox, ulcers, diarrhea, asthma, toothache, jaundice	0.10	0.30	26◆101∎21◆102●103∎104◆66●105∎80◆
75	*Murraya koenigii* (L.) spreng. Rutaceae, HAF-74	Curry leaf	Kari patta	April-June	C	T	P	LE. juice, decoction, infusion, paste; SD; BA. powder	Oral, Topical	Diabetes, diarrhea, skin eruption, rheumatic pain, eye pain, hair tonic	0.07	0.79	26◆101∎21◆102●103∎104◆66●105∎80◆
76	*Veronica polita* Fries* Scharopholariaceae, HAF-75	Grey field speedwell	Veroni	March-May	W	H	A	LE.ST. cooked; LE and ST. decoction; LE. tea, juice	Oral	Indigestion, nerve-tonic, blood purifier, cough	0.10	0.20	26◆101∎21◆102●103∎104◆66●105∎80◆
77	*Datura innoxia* Mill. Solanaceae, HAF-79	Thorn apple	Datura	May-October	W	S	P	SD. paste; WP. powder; LE. decoction, extract; ST. infusion; FR.RT. decoction	Oral, Topical and as Inhale	Piles, asthma, cough, laxative, lice killer, premature ejaculation, sedative and narcotic, rabies	0.07	0.57	26◆101∎21◆102●103∎104◆66●105∎80◆
78	*Solanum nigrum* L. Solanaceae, HAF-76	Night shade	Mako	April-June	W	H	A	LE. decoction, powder cocked, LE. extract; LE and FL. juice; RT. pate; WP. decoction	Oral, Topical, Eye drop	Cuts and wounds, breast cancer, chicken pox, fever, diarrhea, ulcer, piles, cardiac pain, diabetes, sore eyes	0.14	0.83	26◆101∎21◆102●103∎104◆66●105∎80◆
79	*Solanum surattense* Burm.f. Solanaceae, HAF-77	Thorny nightshade	Kundiari	Throughout the year	W	H	P	WP. decoction, cooked; RT. decoction; FR. paste; LE and FR. decoction; RT. decoction	Oral, Topical	High fever, foot cracks, vermifuge, liver tonic, wound healing, rheumatism, kidney stones, asthma	0.17	0.86	26◆101∎21◆102●103∎104◆66●105∎80◆
80	*Withania somnifera* (L.) Dunnel. Solanaceae, HAF-78	Winter cherry	Asgandh	Throughout the year	W	H	P	LE. powder, paste, decoction; RT. powder; WP. powder; FR.FL. powder	Oral, Topical and as Snuff	Malaria, night mare, stomachache, asthma, breast cancer, diabetes, wounds, menstrual flow	0.14	0.86	26◆101∎21◆102●103∎104◆66●105∎80◆
81	*Pterospermum acerifolium* (L.) Willd* Starculiaceae, HAF-80	Maple-leaved Bayer tree	Kanakchanpa	December-July	W/C	T	P	FL. infusion, decoction, paste; BA. powder	Oral, Topical	Anthelminthic, tonic, bleeding piles, body swellings, impotency	0.04	0.56	26◆101∎21◆102●103∎104◆66●105∎80◆
82	*Tamarix aphylla* (L.) Karst. Tamaricaceae, HAF-81	Rukhh	Athel tamarisk	June-October	W	T	P	LE. decoction, poultice, paste; BA. ash	Oral, Topical	Fever, boils and wound healing, eye inflammation, cold and cough	0.08	0.24	26◆101∎21◆102●103∎104◆66●105∎80◆
83	*Cannabis sativa* L. Urticaceae, HAF-83	Marijuana	Bhang	April-September	W/C	S	P	LE. infusion, paste, extract; SD. decoction; LE.SD. juice; WP. powder	Oral, Topical and as Inhale	Diarrhea, constipation, sedative, intoxicant, lice killer, diuretic and laxative, asthma, snake bite	0.08	0.53	26◆101∎21◆102●103∎104◆66●105∎80◆
84	*Lantana camara* L. Verbenaceae, HAF-84	Lantana	Lantana	Throughout the year	W	S	P	FL. extract; RT. extract; LE. sap, juice, paste, decoction	Oral, Topical	Headache, ringworm, injuries, toothache, malaria, rheumatism, cuts and wounds, cold, cough	0.06	0.69	26◆101∎21◆102●103∎104◆66●105∎80◆
85	*Tribulus terrestris* L. Zygophyllaceae, HAF-85	Puncture Vine	Gukhroo	Throughout the year	W	H	A/B	FR. decoction, powder; WP. decoction, powder; LE. paste	Oral, Topical	Dysentery and diarrhea, urodynia, irregular menstruation, wounds, dyspepsia	0.13	0.27	26◆101∎21◆102●103∎104◆66●105∎80◆

^**A**^ Habit/Life forms: W, Wild; C, Cultivated; H, Herbs; S, Shrubs; G, Grass; T, Trees; A, Annual; B, Biennial; P, Perennial

^**B**^ Plant Part(s): LE, Leaf; FR, Fruit; RT, Root; ST, Stem; SH, Shoot; WP, Whole Plant; SD, Seed; FL, Flower; BA, Bark; RH, Rhizome

^C^RFC, Relative frequency of citation; UV, Use value

^**D**^(∎) = Plant with similar use(s); (●) = Plant with dissimilar use (s); (◆) = Plant not reported in previous study

* Plant species, which are newly reported in this study

The life forms of the reported species are mentioned in Table 1. The herbaceous flora constitutes highest contribution (44%) of the reported plant species, which is comparable to Mahmood et al. [2], who reported 54% contribution of wild herbs in medicinal plants used by the local communities of Gujranwala district, Pakistan. The herbaceous species comprise perennial, biennial and annuals (56.5, 1.2 and 27.1%, respectfully).Furthermore, wild and cultivate trees contribute (17 and 10%, respectively), wild grass (8%), wild and cultivate shrubs (9 and 7%, respectively), cultivated herbs (4%) and cultivated grasses (1%) in descending order (S2 Fig). These findings were in consistence with previous reports [19,49,52–56]. The frequent use of herbs by indigenous communities may be due to their accessibility and high efficacy in the treatment of diseases compared to other life form [57–60].It is well-known that the medicinal plants having perennial nature require prolonged period of growth i.e. about 6–8 years depending on the type of plant species. Therefore, the perennial life cycle is more prominent in medicinal plant species than annual [22,61–63].

### Plant part(s)used, mode of preparation and application

**Fig 3:**
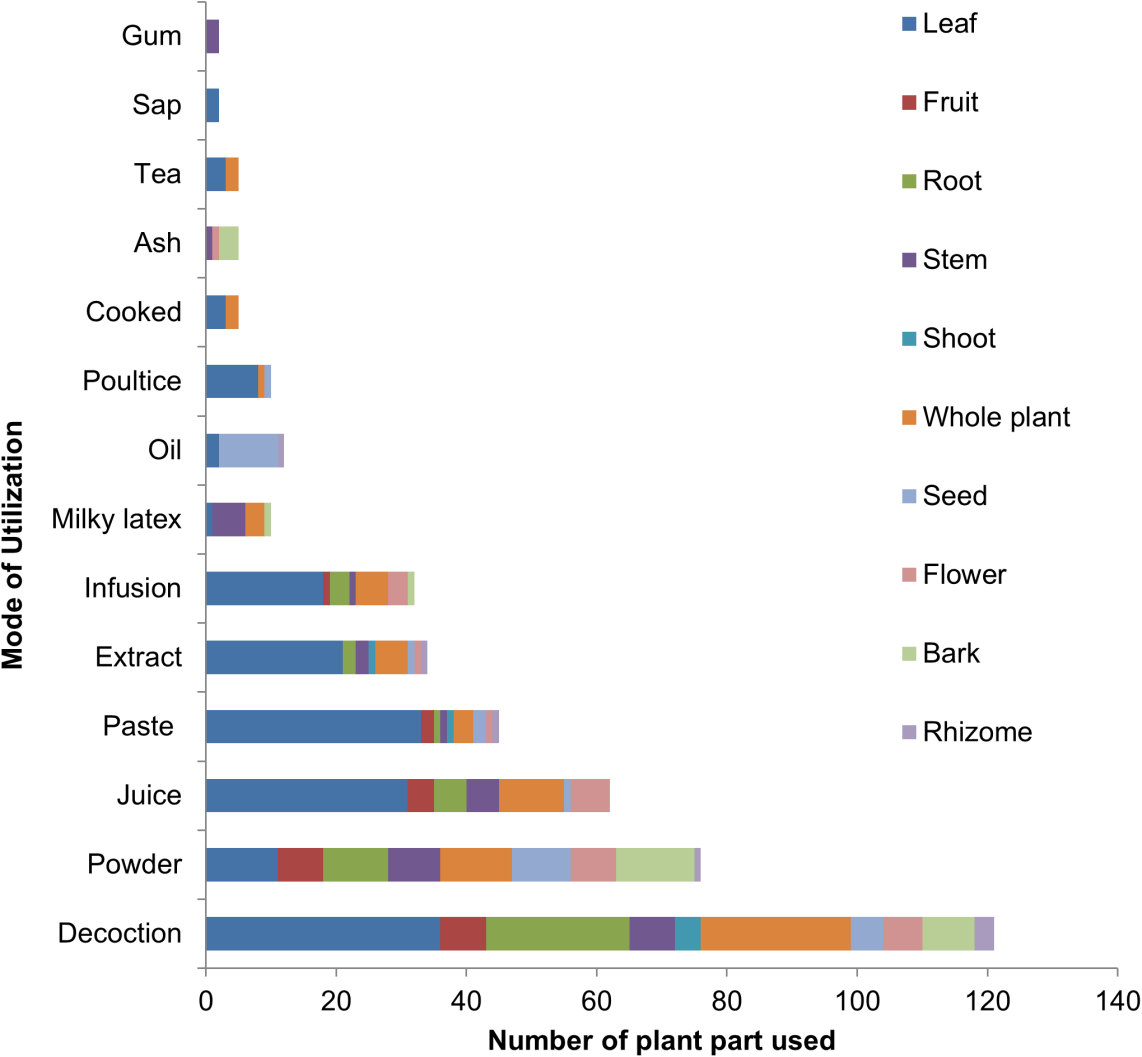
Proportional contributions of plant part in herbal preparations.

Even though all plant parts are significant in the treatment of different ailments; nevertheless in the present study as shown in Fig 3, leaves were the most commonly utilized plant part with38% application in traditional medicinal recipes, followed by whole plant (14%), root (10%), stem (8%), fruit, seed, flower (7% each), bark (5%), shoot and rhizome (2% each). Bradacs et al. [64] and Leto et al. [65]also reported leaves as commonly utilized plant part in herbal medicine used by the inhabitants of islands and Italy. It has been reported that the use of leaves is better for the survival of medicinal plants collected by herbalists compared to the collection of whole plant, roots and stem, which may cause severe threat to local flora [66].Even though some plant species such as *Calotropis procera*, *Croton sparsiflorus*, *Datura innoxia*, *Euphobia spp*., *Lantana camara*, *Solanum spp*. and *Ranunculus sceleratus* are consider as poisonous; however used to treat various ailments by local inhabitants. It has been reported that plant species with potent bioactive compounds are often characterized as poisonous and medicinal as well, and a beneficial or an adverse result may depends on method of drug preparation and utilization [67]. It was observed that inhabitants of the study area use above mentioned species in modest quantity, therefore no toxic effect was mentioned by respondents.

Local inhabitants of the study area use different methods i.e. decoction, extract, juice, powder, paste, infusion, poultice, tea, and ash etc. to prepare a recipe for the treatment of various ailments (Fig 3). Decoction was the most common method of drug preparation (121 applications), followed by powder, juice, paste, extract and infusion (76, 62, 45, 34 and 32 applications, respectively). Whereas, milky latex and oil were used in 12 applications each, poultice in 10 applications, cooked food, tea and ash with 5 applications each and plant sap with 2 applications. Such a wide array of preparation methods has also been reported previously from different parts of Pakistan [2,23,68,69] and in other countries [49,70–74]. The traditional healers of the study area also use more than two plant species along with other ingredients i.e. milk, honey, egg, butter, salt, sugar and water etc. The widespread use of decoction and powder in the study area is comparable to Mahmood et al. [2], Ahmad et al. [68] and Boudjelal et al. [75] who reported decoction as a most commonly utilized method of preparation followed by powder. Decoction is used as one of the major practices to prepare drug in traditional healing system, because it is easy to make by mixing with tea, water or soup [76,77].While making decoction, plant material is boiled in water until volume of the water reduced to one-fourth of its original volume [69], whereas crude extract is obtained by squeezing or crushing the plant parts [78].

As far as mode of administration is concerned, around 69% medications were taken orally (S3 Fig), followed by administered topically (24%), as eye drops and gargle (2% each), and as toothbrush, inhale and anal application (1% each). These findings were comparable to earlier reports [45,79,80]. Moreover, some herbal preparations were used for bathing, sniff and as eardrops. The medicinal plant species used as sniff are burnt to inhale fumes. Likewise, some plant parts were just crushed and smelled. Similar modes of applications were reported in Gujranwala [2]. The leaf and bark of some plant species are boiled and decoction is used to take bath against body pain. However, the trend of herbal bath is declining due to changing life style and now days only the leaves of *Zizyphus mauritiana* are used to bath the dead bodies. For topical use, direct application of paste, poultice or medicated oil are common, which are mostly used to treat skin infections, cuts, wounds, scorpion bites, rheumatism headache and body pain.

### Informant consensus factor (ICF)

To calculate ICF, the reported ailments were first classified into 11 different disease categories on the basis of their use reports (Table 2).Among three major disease categories, dermatological disorders were dominated with 111 use-reports, followed by glandular complaints and respiratory diseases (76 and 52 use-reports, respectively) as mentioned in S4 Fig. Around 79.1% plant species were used to treat dermatological ailments, followed by gastrointestinal track (GIT) disorders, glandular complaints, respiratory diseases, ENEM diseases, cardiovascular disorders, urinary problems, muscle and skeletal disorders, sexual diseases and nervous disorders (76.7, 62.8, 47.7, 33.7, 25.6, 24.4, 23.3, 22.1 and 14%, respectively). These findings signify that dermatological and GIT disorders are prevalent in the study area. Similar findings have already been reported [53,64,65,81–86]. However, Kadir et al. [45] and Singh et al. [57]described more number of species to treat gastrointestinal diseases compared to dermatological ailments. The ICF value of different disease categories was ranged from0 (nervous disorder) to 0.39 (GIT diseases) The average ICF value for all categories was 0.16, which was similar to previous studies carried out in Pakistan [23,48].

**Table 2:** Informant consensus factor (ICF) of reported plant species against various ailments.

Category of Diseases	Number of use reports	% age of use reports	Number of taxa used	% age of taxa	*ICF
GIT diseases	107	21.9	66	76.7	0.39
Respiratory diseases	52	10.6	41	47.7	0.22
Muscles and Skeletal disorders	23	4.7	20	23.3	0.14
Urinary disorders	22	4.5	21	24.4	0.05
Sexual diseases	20	4.1	19	22.1	0.05
Glandular disorders	76	15.5	54	62.8	0.29
Ear, Nose, Eyes and Mouth (ENEM) disease	31	6.3	29	33.7	0.07
Nail, Skin and Hair disorders	111	22.7	68	79.1	0.39
Nervous disorders	12	2.5	12	14.0	0.00
Cardiovascular disorders	23	4.7	22	25.6	0.05
Body energizers	11	2.2	10	11.6	0.10

*ICF = Informant Consensus Factor

### Relative frequency of citation (RFC) and use value (UV)

The RFC and UV indices are applied to select potential plant species for further pharmacological study and recommendation in drug development. The relative frequency citation (RFC) index authenticates the frequency of citation of a medicinal plant species used for various ailments. The RFC of the reported species ranged from 3to 17% (Table 1). However, on average, the RFC was9%. The highest RFC was calculated for *Solanum surattense* (0.17), *Eclipta alba* (0.15) and *Triticum aestivum* (0.15).The positions of these plant species correspond to the fact that they were reported by maximum number of informants, therefore having high frequency of citation (FC).

The use value (UV) index demonstrates the relative importance of plant species and families for a population [87]. In the present investigation, the UV of the reported medicinal plant species varied from 0.14 to 0.86 (Table 1). The highest UV was calculated for *Solanum surattense* and *Withania somnifera* (UV = 0.86, for each), *Cyperus rotundus* (UV = 0.84) and *Solanum nigrum* (UV = 0.83). These findings demonstrate the extensive use of above mentioned species in the treatment of various ailments by local inhabitants/healers and the consciousness of indigenous peoples, which makes such medicinal plants, the first choice to treat a disease. The lowest UV of *Anethum graveolens*, *Brassica rapa* and *Chrozophora tinctoria* may be due to less accessibility and minimum ethnobotanical uses. The results of UV in the present study were comparable with previously reported from Gujranwala, Pakistan [2].It has been reported that *S*. *surattense* exhibits hepato-protective[88], anti-asthmatic[89],antioxidant, anthelmintic, antimicrobial[90], wound healing[91], diuretic[92] and antipyretic [93] properties.Devi et al. [94]evaluated antibacterial, anti-fungal and antitumor properties of *W*. *somnifera* while its root has been used since long time for both sexes and even during pregnancy[95]. Another medicinal plant, *C*. *rotundus* has been studied against skin disease [96]. Recently, anticancer activity of *S*. *nigrum* has been reported by Lai et al. [97]. These findings confirm high RFC and UV of these plant species in the study area.

### Relative popularity level (RPL)

Our 201 informants cited 85 plant species for 11 different disease categories. Of them, 32 species as given in Table 3, received more consideration by informants; therefore included for further discussion. The correlation between number of informants citing a particular plant species and the number of application used is given in S5 Fig, whereas S6 Fig indicates relationship among the numbers of informants claimed use of certain plant species for a particular disease. For species cited by 6 to 25 informants, number of uses per plant increases linearly with the increase in number of informants i.e. correlation coefficient *r* = 0.10 (S5 Fig). The average number of uses for plant species cited by 26 or more informants does not increase with the increased number of informants. 26 plant species, which were cited by few to 25 informants were declared unpopular, whereas the 6 plant species mentioned by 26 informants or more were classified as popular. The separating line between the popular and unpopular groups falls at the point where average number of uses per plant ceases to increase with further increase in the number of informants. *Solanum surattense* (Solanaceae), *Triticum aestivum* (Poaceae), *Solanum nigrum* (Solanaceae), *Withania somnifera* (Solanaceae), *Ranunculus sceleratus* (Ranunculaceae) and *Calotropis procera* (Asclepiadaceae) were the popular plant species with 1.0 RPL value. The high popularity of these plant species might be attributed to their high efficacy and the awareness of indigenous peoples which specifies their use as herbal medicine. This is the first baseline study on the indigenous knowledge of local peoples regarding the use of popular plant species for a particular ailment. These findings were in consistent with previous studies on the status of medicinal plants among Bedouins of Negev district [43] and medicinal plant survey in Palestinian area [44]. In these studies *Alhagimaurorum* (urinary disorder) and *Tamarixaphylla* (eye problem) were reported as unpopular plant species due to low RPL.

**Table 3:** Highly utilized species of the study are along with FL, RPL and ROP.

S. No	Species name	N	NA	Major ailment	Np	FL	RPL	ROP
1.	*Solanum surattense*	52	8	Kidney stones	44	84.6	1.00	85
2.	*Triticum aestivum*	42	6	Late puberty	33	78.6	1.00	79
3.	*Solanum nigrum*	39	12	Cardiac pain	30	76.9	1.00	77
4.	*Withania somnifera*	34	8	Stomachache	34	100	1.00	100
5.	*Ranunculus sceleratus*	28	6	Urinary incontinence	28	100	1.00	100
6.	*Calotropis procera*	26	8	Wound healing	20	76.9	1.00	77
7.	*Cyperus rotundus*	25	6	Hypersplenism	11	44.0	0.96	42
8.	*Achyranthes aspera*	23	8	Dysmenorrhea	10	43.5	0.88	38
9.	*Chenopodium album*	23	7	Stomachache	7	30.4	0.88	27
10.	*Ficus religiosa*	22	6	Skin ulcer	6	27.3	0.85	23
11.	*Malva parviflora*	22	8	Cough and bronchitis	7	31.8	0.85	27
12.	*Sonchus asper*	22	5	Constipation	6	27.3	0.85	23
13.	*Acacia nilotica*	21	8	Hyperglycemia	8	38.1	0.81	31
14.	*Anethum graveolens*	21	4	Indigestion	3	14.3	0.81	12
15.	*Boerhavia diffusa*	21	5	Asthma	6	28.6	0.81	23
16.	*Eucalyptus camaldulensis*	21	7	Sore throat	6	28.6	0.81	23
17.	*Launaea procumbens*	20	4	Ringworm	4	20.0	0.77	15
18.	*Veronica polita*	20	4	Nerve tonic	4	20.0	0.77	15
19.	*Brassica rapa*	19	2	Skin edema	3	15.8	0.73	12
20.	*Oxalis corniculata*	19	8	Jaundice	7	36.8	0.73	27
21.	*Ziziphus nummularia*	19	6	Constipation	5	26.3	0.73	19
22.	*Anagallis arvensis*	18	4	Skin ulcer	4	22.2	0.69	15
23.	*Euphorbia dracunculoides*	18	5	Headache	5	27.8	0.69	19
24.	*Trifolium resupinatum*	18	6	Skin sores	7	38.9	0.69	27
25.	*Cannabis sativa*	17	9	Nervetonic	9	52.9	0.65	35
26.	*Tamarix aphylla*	17	6	Eye inflammation	4	23.5	0.65	15
27.	*Alhagi maurorum*	15	8	Kidney stone	8	53.3	0.58	31
28.	*Melia azedarach*	14	7	Malarial fever	11	78.6	0.54	42
29.	*Cirsium arvense*	12	5	Peptic ulcer	4	33.3	0.46	15
30.	*Rumex dentatus*	11	4	Ecezma	7	63.6	0.42	27
31.	*Kochia indica*	8	3	Heart tonic	3	37.5	0.31	12
32.	*Malvaviscus arboreus*	6	3	Diarrhea	4	66.7	0.23	15

N. number of total informants, NA. Number of ailments, Np. No. of informants who reported use of species, FL. Fidelity level, RPL. Relative popularity level, ROP. Rank order priority

### Fidelity level (FL)

The fidelity level (FL) of the 32 most important plant species ranged from 14.3 to 100% (Table 3). In general, the high FL of a species indicates the prevalence of a specific disease in an area and the utilization of plant species by the inhabitants to treat it [23,98]. *Withania somnifera* and *Ranunculus sceleratus* depicted 100% FL against stomach and urinary disorders, respectively. The fidelity levels calculated for *Solanum surattense*, *Triticum aestivum*, *Melia azedarach*, *Solanum nigrum* and *Calotropis procera* to treat kidney stones, late puberty, malarial fever, cardio-vascular diseases, and wound healing were 84.6, 78.6, 78.6, 76.9, 76.9%, respectively. The plant species with100% FL for instance *W*. *somnifera* have also been reported as chemo-preventive against stomach and skin carcinogenesis [99]. In another study, the *R*. *sceleratus* was reported as antiurolithiatic agent to treat urinary disorder [100].

### Rank order priority (ROP)

The Rank order priority (ROP) index is used to rank appropriately the plant species with different FL values. The resultant RPL values given in S5 Fig were used as correction factor to adjust the FL values. The ROP values are thus obtained are given in Table 3. Of the 32 species, only eight species attained ROP above 50.This is probably due to decreasing popularity of herbal medicines among the local communities of the study area. Based on ROP value *Withania somnifera* and *Ranunculus sceleratus* were widely utilized species with ROP = 100 for each. The other plant species with significant ROP were: *Solanum surattense*, *Triticum aestivum*, *Solanum nigrum*, and *Calotropis procera* (86, 79, 77 and 77, respectively). The ROP values reported for medicinal plants used by Bedouins community in Negev district [43] and in Palestinian area [44] were comparable to present findings. However, *Alhagim aurorum* (ROP = 31) and *Tamarix aphylla* (ROP = 15), were used to relieve urinary system disorder and eye disease, respectively by the inhabitants of the study area.

### Novelty and future impact

Present study is the first document on ethnobotanical uses of 85 medicinal plant species used by the inhabitants of Hafizabad district, Punjab-Pakistan. The current ethnomedicinal uses of reported plant species were compared previous studies conducted in Pakistan and other areas [21,26,66,80,101–105] as shown in Table 1, to find the novelty index. Approximately,15% medicinal uses of reported species were similar, whereas 6% were dissimilar to previous reports. However, 79% medicinal uses were new in the present study. Moreover, medicinal uses of plant species reported in neighboring areas i.e. from Layyah district, Punjab province, Pakistan [21] showed more resemblance compared to those documented in other areas. The data collected from the study area reveal considerable difference in plant parts used, mode of herbal preparation and its utilization as reported from other regions.

Some of the newly documented medicinal uses and relevant plant species include: *Anethum graveolens* (indigestion), *Chrozophora tinctoria* (stomachache), *Cirsium arvense* (to heal wounds), *Euphorbia prostrate* (diarrhea and dysentery), *Ficus benjamina* (stomach disorder), *Jasminum officinale* (fever), *Kochia indica* (toothache), *Malvastrum tricuspidatum* (sores & wounds), *Morus nigra* (asthma), *Parthenium hysterophorus* (constipation), *Prosopis juliflora* (kidney stones), *Pterospermum acerifolium* (bleeding piles), *Ranunculussceleratus* (asthma), *Setaria glauca* (skin disease), *Sorghum halepense* (indigestion), *Trifolium resupinatum*(sore throat) and *Veronica polita* (indigestion). The plant species with new medicinal uses and high RPL value could be studied further to screen bioactive compounds and their pharmacological activities to introduce novel drugs.

## Conclusion

Present survey revealed that a number of medicinal plant species are used by indigenous people of the study area to treat various ailments. The indigenous community still relies on traditional medicine although; the modern health-care services are available, which indicates the significance of plant based traditional recipes. Our findings provide baseline data to establish a connection between the traditional health practioners and scientific communities, which could be substantial in novel drug discovery. Furthermore, ethnobotanical data is of significant value for conservation managers and policy makers for sustainable management of medicinal plant species, which are under threat due to over exploitation. The high RPL value of medicinal plant species is an indication of their preference by local inhabitants to treat particular ailments. Therefore such popular plant species could be further analyzed for bioactive constituents, *in vivo/in vitro* biological activities, which may leads to new and potential drugs.

## Supporting information

S1 Fig Dominant families of medicinal plants. (TIF)

S2 Fig Habit wise categorization of medicinal plant species. (TIF)

S3 Fig Percentage modes of application for traditional recipes. (TIF)

S4 Fig Disease categories and number of use reports. (TIF)

S5 Fig Correlation between number of informants citing a particular plant species and the number of application used. Numbers represent the plant names as appear in Table 3.
(TIF)

S6 Fig Relationship between the numbers of informants claimed use of certain plant species for a particular disease. Numbers represent the plant names as they appear in Table 3.
(TIF)
