# Supplementary figures and images for: An ethnobotanical survey of indigenous medicinal plants in Hafizabad district, Punjab-Pakistan
Source: PLoS One. 2017 Jun 2;12(6):e0177912. doi: 10.1371/journal.pone.0177912 (PMC5456064; doi:10.1371/journal.pone.0177912)

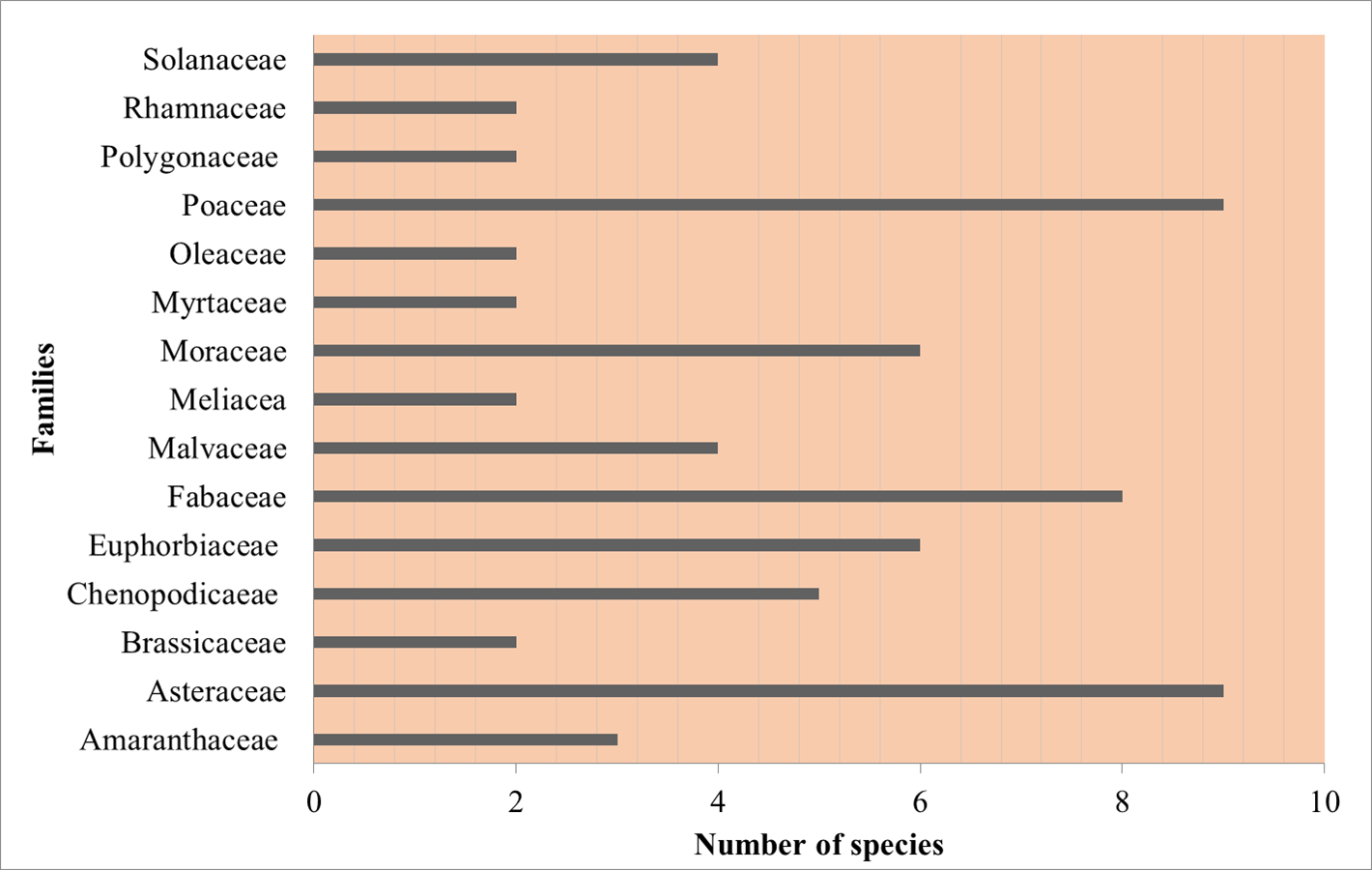

Supplement: S1 Fig — (TIF) [file pone.0177912.s001.tif]

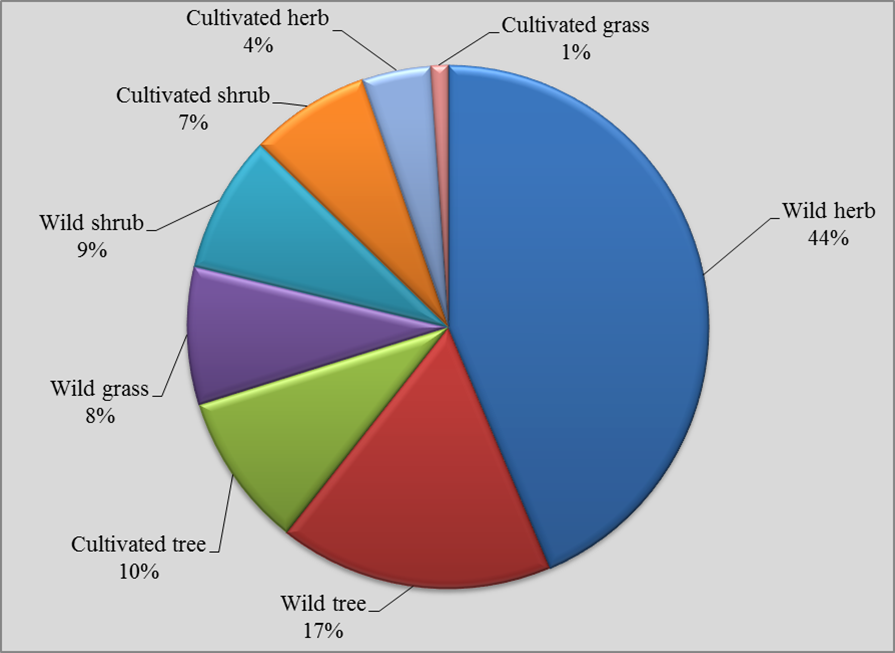

Supplement: S2 Fig — (TIF) [file pone.0177912.s002.tif]

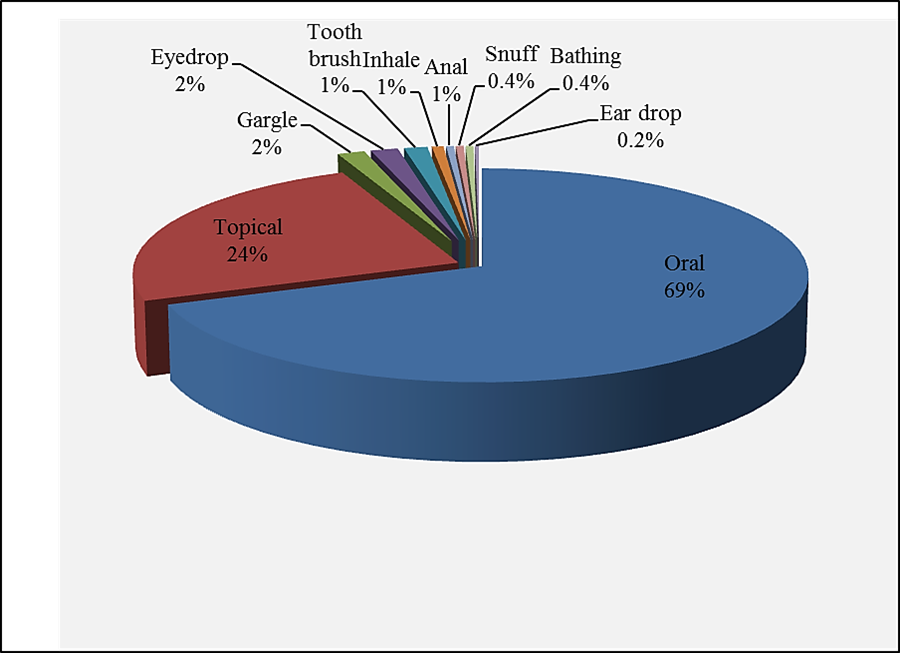

Supplement: S3 Fig — (TIF) [file pone.0177912.s003.tif]

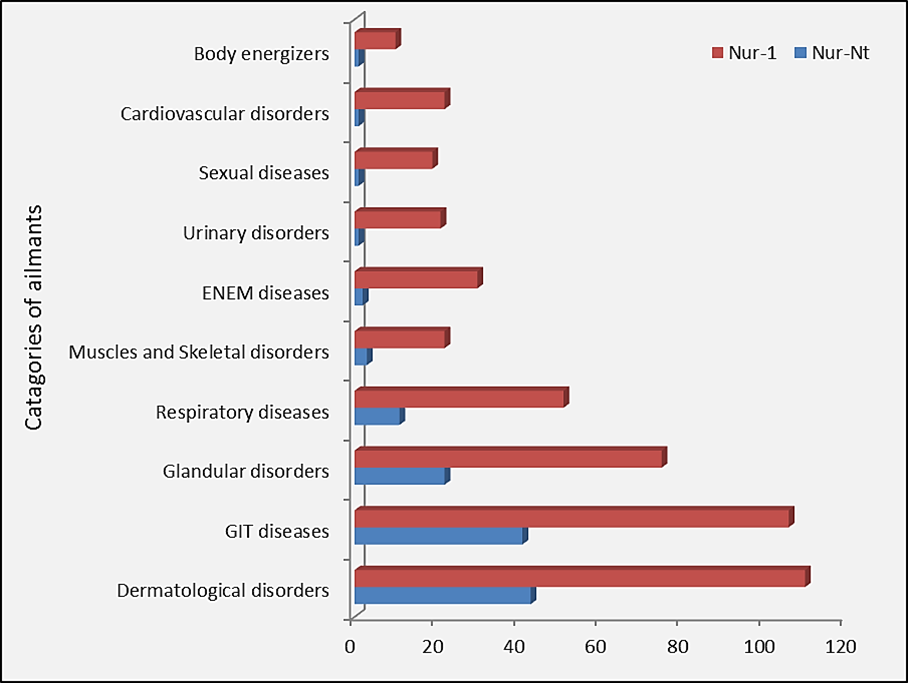

Supplement: S4 Fig — (TIF) [file pone.0177912.s004.tif]

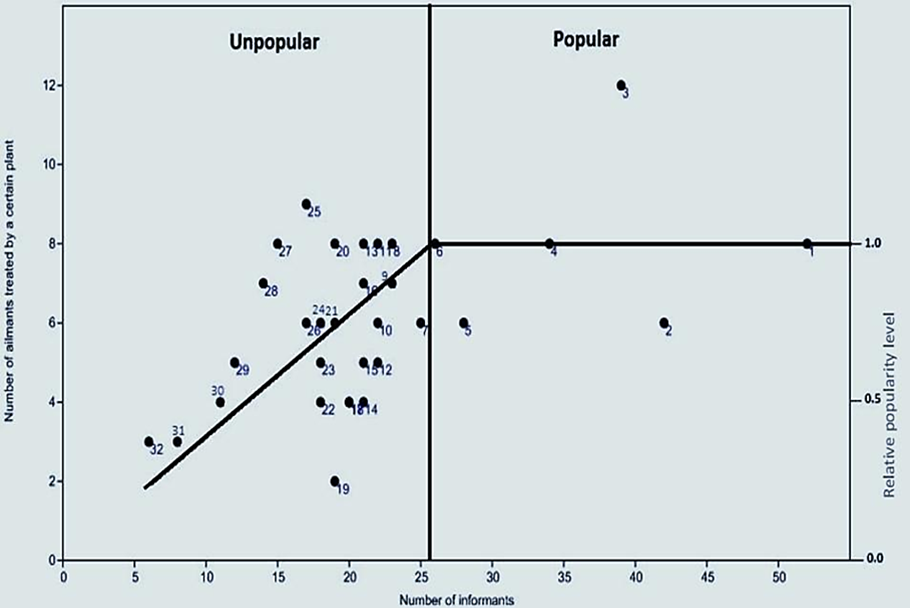

Supplement: S5 Fig — Numbers represent the plant names as appear in Table 3. (TIF) [file pone.0177912.s005.tif]

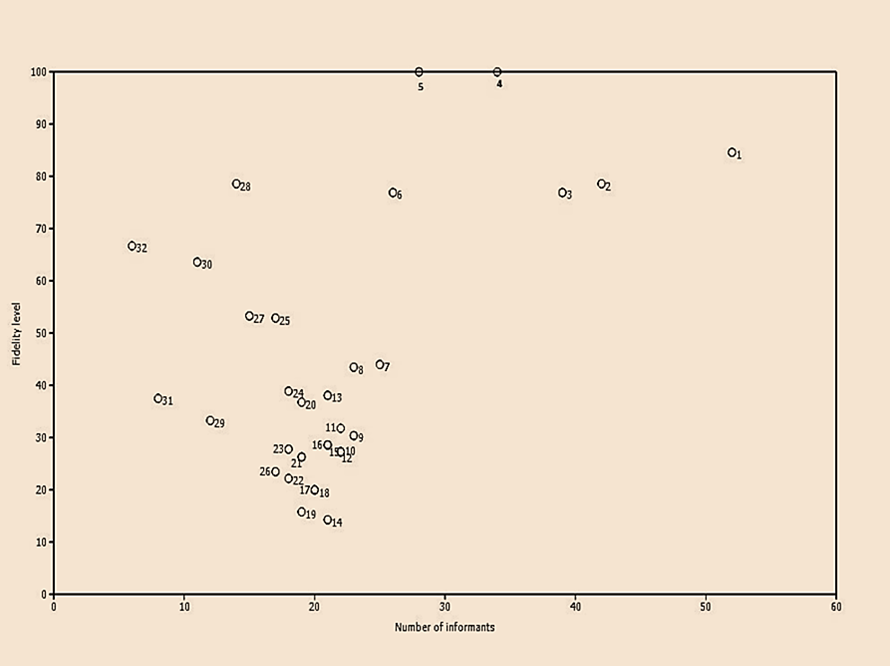

Supplement: S6 Fig — Numbers represent the plant names as they appear in Table 3. (TIF) [file pone.0177912.s006.tif]
